# Echocardiography Differentiates Lethally Irradiated Whole-Body From Partial-Body Exposed Rats

**DOI:** 10.3389/fcvm.2018.00138

**Published:** 2018-10-16

**Authors:** Taeko Inoue, Janice A. Zawaski, Vivien Sheehan, Celeste Kanne, Alireza Paikari, Caterina C. Kaffes, Poonam Sarkar, Omaima M. Sabek, M. Waleed Gaber

**Affiliations:** ^1^Hematology-Oncology Section, Department of Pediatrics, Dan L. Duncan Cancer Center, Baylor College of Medicine, Houston, TX, United States; ^2^Department of Surgery, Houston Methodist Hospital Research Institute, Houston, TX, United States; ^3^Department of Molecular Physiology and Biophysics, Baylor College of Medicine, Houston, TX, United States

**Keywords:** echocardiography, magnetic resonance imaging, radiation, rheology, anemia, dense red blood cells

## Abstract

**Background:** Acute radiation syndrome (ARS) affects morbidity and mortality dependent on the amount of body exposed. We propose the use of echocardiography (EC) to differentiate between survivors and non-survivors by measuring changes in cardiac function (CF) and pulmonary arterial function (PAF). We also investigate the role of rheology in our observed changes.

**Methods and Results:** Rats were irradiated to the whole body (WB) or partial body with two-legs shielded (2LS) at a lethal dose of 7.5Gy. EC and magnetic resonance imaging were performed, and rheological measurements conducted. Only 2LS survived past 12-days post-exposure and their CF and PAR were not significantly different from baseline. WB was significantly different from both baseline and 2LS in stroke volume (*P* < 0.05), velocity time integral (VTI; *P* < 0.05) and pulmonary artery acceleration time (PAAT; *P* < 0.05). Differences were identified as early as six-days post-exposure, where VTI and PAAT were significantly (*P* < 0.05) decreased in WB versus baseline but only PAAT was different from 2LS. Blood viscosity was significantly lower in the WB versus baseline and 2LS (*P* < 0.0001). WB exhibited a significant rise in dense red blood cells versus baseline (*P* < 0.01) and 2LS (*P* < 0.01). Cell-free hemoglobin, a contributor to pulmonary artery hypertension and vasculopathy, was significantly elevated in WB vs. sham.

**Conclusions:** Non-invasive and readily available imaging can be used to identify critically affected victims. Our findings point to heart failure as one possible cause of death in WB exposed animals, potentially exacerbated by rheological, hemolytic, and pulmonary factors, and the importance of developing radiomitigators against cardiac ARS mortality.

## Introduction

Acute radiation syndrome (ARS) results from whole or partial body exposure to lethal doses of radiation and has been mainly characterized by hematopoietic, gastrointestinal, and cardiovascular-central nervous system damage, in order of increasing severity of damage. In the aftermath of a nuclear detonation, healthcare facilities will be overwhelmed with radiation victims who are suffering with symptoms of ARS. Difference in the extent of body radiation exposure (RE) will result in marked differences in the victim's survival probability, and in their acute and late prognosis, resulting in some requiring immediate attention while others could be evaluated at a later time. To date, there is no consensus on a standardized triaging and assessment paradigm (1). The main health concerns in the acute phase, following RE, have focused on hematopoietic and gastrointestinal symptoms. However, long-term epidemiological studies of survivors of the Hiroshima and Nagasaki atomic bombing show that survivors have a higher risk of circulatory disease such as stroke and heart diseases, with 17% of non-cancer related deaths in A-bomb survivors related to heart disease (1, 2). While several studies document the long-term effect of RE on cardio-pulmonary function there are none, that we are aware of, that address the immediate or short-term effects. Our study is new in that it addresses this gap of knowledge.

Cardiac function (CF) is one of the primary indicators of morbidity and is routinely monitored in emergency wards in critically ill patients. Imaging, using echocardiography (EC) and/or Magnetic resonance (MR), plays an important role in the diagnosis of cardiac problems and in guiding treatment (3, 4). Despite some efforts to include imaging in preparedness plans, such as the inclusion of diagnostic imaging capabilities in the Radiation Emergency Assistance Center (5), it is not as yet an integrated tool in emergency response to a nuclear incidence (6). Our work addresses this deficit identifying imaging differences that can potentially diagnose and separate those critically affected victims from those that are at lower risk based on their cardiac function differences.

In hematological diseases, such as in anemia or sickle cell disease, rheological changes in viscosity, hematocrit, and cell deformability have been shown to affect blood flow, vascular resistance, tissue perfusion, and eventually cardiac function (7). Acutely, the main difference between whole and partial body exposed subjects is in the accelerated recovery of the blood constituents of those that are partially shielded and in their significantly improved survival (8, 9). In this paper we investigate the possible link between cardiac function and rheological changes post RE. We will measure the hematocrit to viscosity ratio at high and low shear rates, to model oxygen carrying capacity in the venous and arterial circulation (10, 11), and the percent dense red blood cells (%DRBC), which are red cells with a density>1.11 mg/mL; they are typically the result of cellular dehydration, and are less deformable and more likely to hemolyze (12). We will measure extracellular, or free hemoglobin using the ADVIA hematology analyzer, which measures hemoglobin two ways: total hemoglobin colormetrically, and intracellular hemoglobin flow cytometrically.

In this study, we hypothesize that it is possible to use pulmonary arterial function (PAF) and CF; in the first few days post-RE, to diagnose those that are severely affected. We will demonstrate that RE causes pulmonary arterial dysfunction, changes in CF via alterations in cardiac morphometry, and that body shielding is cardioprotective. We will investigate the role of blood rheology in our observed changes.

## Methods

### Experimental design

Male Sprague Dawley rats (Envigo, Indianapolis, IN) 7–8 weeks of age were used. EC and MR imaging (MRI) was carried out on rats at baseline prior to RE. Cardiac function on the same animal using both EC and MRI was assessed on days 8–9 post-RE. Pulmonary artery function was assessed using US at days 6, and 10 post-RE. The euthanasia criterion was set at a 20% reduction in weight and/or morbidity (Immobility, balance issues, huddled posture, inability to eat, ruffled fur, hypothermia, labored breathing). All animals were used in accordance to the guidelines set by the Institutional Animal Care and Use Committee at Baylor College of Medicine.

### Radiation paradigm

We utilized the RS-2000 x-ray (160 kVp, 25 mA) source irradiator (RadSource, Suwanee, GA). Rats were anesthetized with 3% isoflurane and oxygen and divided among groups; exposed to only isoflurane (SHAM), a 7.5 Gy lethal dose of whole body radiation (WB), or whole body radiation with 2-legs shielded (2LS). Post-RE, rats were monitored and weighed on a regular basis.

### Cardiac MRI

Cardiac function was assessed in all rats using a 9.4 T Bruker BioSpec Avance spectrometer (Bruker BioSpin, Billerica, MA) with a 21 cm horizontal bore, 75 mm resonator. A single slice through the long axis of the left ventricle was delineated using a retrospectively gated IntraGate FLASH protocol that gave temporal information on diastolic and systolic events, and a series of scans were done through the short axis of the heart. The parameters were respectively as follows: TE = 2.948 and 1.912 ms, TR = 8 and 40 ms, # of repetitions = 300 and 100, matrix size = 256 × 256, FOV = 6 × 6 cm, 1–1 and 5–1 mm thick slices with 3 mm interslice distance for the short axis scans and a 5 m 9 s 200 ms and 8 m 37 s 0 ms scan times. The short axis scans were done perpendicularly through the long axis of the heart and each slice consisted of 10 chronological time points ranging from systole and diastole. Three rounds of this scan were done, each time shifting the slices by 1 mm in order to encompass the entire volume of the left ventricle. After data acquisition, IntraGate scans through the short axis of the heart were analyzed using Amira (FEI Visualization Sciences Group, Burlington, MA) software.

### Echocardiography

Echocardiography of all rats using a Vevo 770 High Resolution *in vivo* Micro Imaging System (VisualSonics, Ontario, Canada). RVM 710B 12–38 MHz scan head was utilized for left ventricular assessment, the heart rate was maintained in the range of 350 to 450 beats/min. The rats were anesthetized and kept under 2% isoflurane and oxygen during the procedure. Fur was depilated in regions above the heart and EcoGel 100, an ultrasound gel (Eco-Med Pharmaceuticals Inc., Ontario, Canada), was placed on the chest. The left ventricle was located in B-mode and a cross-section through the short axis of the left ventricle clear of papillary muscles was selected in M-mode and a time-lapse of left ventricular movement was recorded for each rat. Blood flow through the pulmonary artery was measured using power Doppler mode. Ultrasound data was analyzed using the Vevo 770 measurement software. LVID (left ventricular inner dimension), and LVOD (left ventricular outer dimension) were traced and stroke volume calculated from the difference between end-diastolic and end-systolic volumes. Cardiac output is given by the product of stroke volume and heart rate, and ejection fraction was measured as the ratio of stroke volume to end-diastolic volume. PAAT (pulmonary artery acceleration time) and VTI (velocity time integral) measurement tools were measured from the time of systolic flow to peak pulmonary outflow velocity, and by tracing the outer edge of the outflow profile, respectively.

### Rheology

All measurements were made using whole blood within 2 h of blood draw at day nine post radiation exposure. Blood viscosity was measured with a cone and plate viscometer (Brookfield DVII+, with CPE40 spindle) at 37°C. Blood viscosity was measured at two shear rates, 45 s^−1^ and 225 s^−1^, to model venous and arterial blood flow, respectively, and at native hematocrit. Viscosity measurements were performed according to guidelines for hemorheological laboratory techniques (13). Ektacytometry was performed using a RheoScan-D (Rheomeditech, Republic of Korea), as previously described (14).

### ADVIA 120 analysis

Hemoglobin concentration (Hb), red blood cell count (RBC), white blood cell (WBC), platelet count, mean corpuscular volume (MCV), mean corpuscular hemoglobin concentration (MCHC), absolute reticulocyte count (ARC), and hemoglobin distribution width (HDW), were determined using a Siemens ADVIA 120 Hematology Analyzer. Hemoglobin (Hb) delta, or cell free Hb, was measured using the ADVIA, total Hb- cellular Hb, and adjusted for reduced erythropoiesis by dividing Hb delta by total Hb.

### Statistical analysis

Statistical analysis was done using GraphPad software (Prism, La Jolla, CA). One–way analysis of variance was run at each time point comparing between baseline, 2LS and WB irradiated rats *post hoc* comparisons were made using a Tukey's test. A *p*-value equal to or < 0.05 was considered to be statistically significant mean +/− standard deviations are shown.

## Results

### Survival

**Figure 1:**
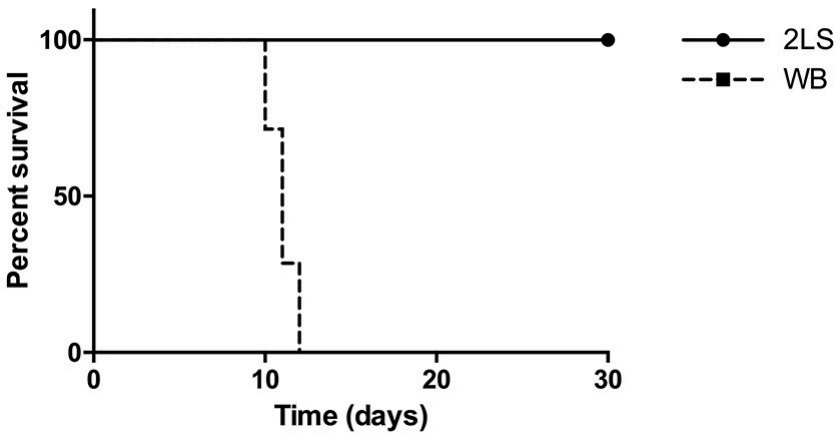
Radiation exposure (RE) affects percent survival of whole body (WB) and two leg shielded (2LS) groups. The WB group meets the euthanasia criteria on average by 10 days post-RE, while the 2LS group survives past the thirty-day time point. N = 6-7.

As shown in (Figure 1), 7.5 Gy of WB exposure of irradiation is lethal to rats. The euthanasia criteria was met between day 10 and 12 post-RE. However, none of the 2LS rats met the criteria during the month post RE in which they were monitored. This study confirms the protective effect of sparing bone marrow.

### Cardiac function

**Figure 2:**
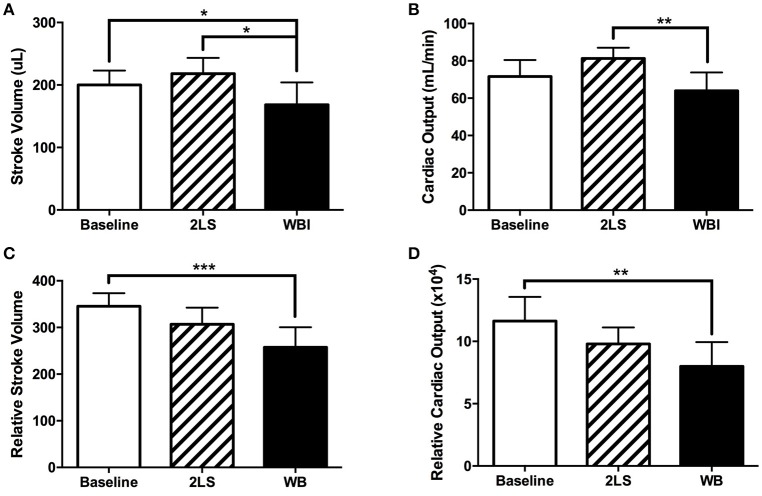
Acute effects of radiation exposure (RE) on cardiac function (8-9 days post exposure). (Top row measurements using echocardiography). **(A)** Stroke volume significantly decreased in the whole body (WB) irradiated group compared to both baseline and two legs shielded (2LS) group. **(B)** Cardiac output significantly decreased in the WB compared to the two legs shielded (2LS), but not the baseline and group. (Bottom row measurements using MRI). **(C)** While relative stroke volume in the WB was significantly lower than baseline, it was not different from 2LS. **(D)** Relative cardiac output was significantly reduced in the WB group. *N* = 5–12, ^*^p < 0.05, ^**^p < 0.01, ^***^p < 0.001.

The standard clinical practice is to assess cardiac function using EC and/or MR imaging. We utilized both measurements to investigate LV function in rats, post-RE. At day eight post-RE we observed a significant decrease in stroke volume in the WB group compared to baseline, as seen in Figures 2A–C, in both EC (*P* = 0.0427) and MR (*P* = 0.0007) measurements. However, only the EC stroke volume measurement showed a significant difference between the 2LS and WB (*P* = 0.0114). RE caused a significant decrease (*P* = 0.0044) in cardiac output in the WB compared to the 2LS, but not baseline when measured with EC (Figure 2B). MR measurements showed a significant difference (*P* = 0.0073) between WB and baseline but not 2LS (Figure 2D). Further, RE caused an increase in ejection fraction compared to baseline but only WB was significantly higher (*P* = 0.0228), see Supplement Table 1. Our repeatability measurements demonstrated consistent results with no significant differences between groups, see Supplement Table 2.

### Pulmonary artery

**Figure 3:**
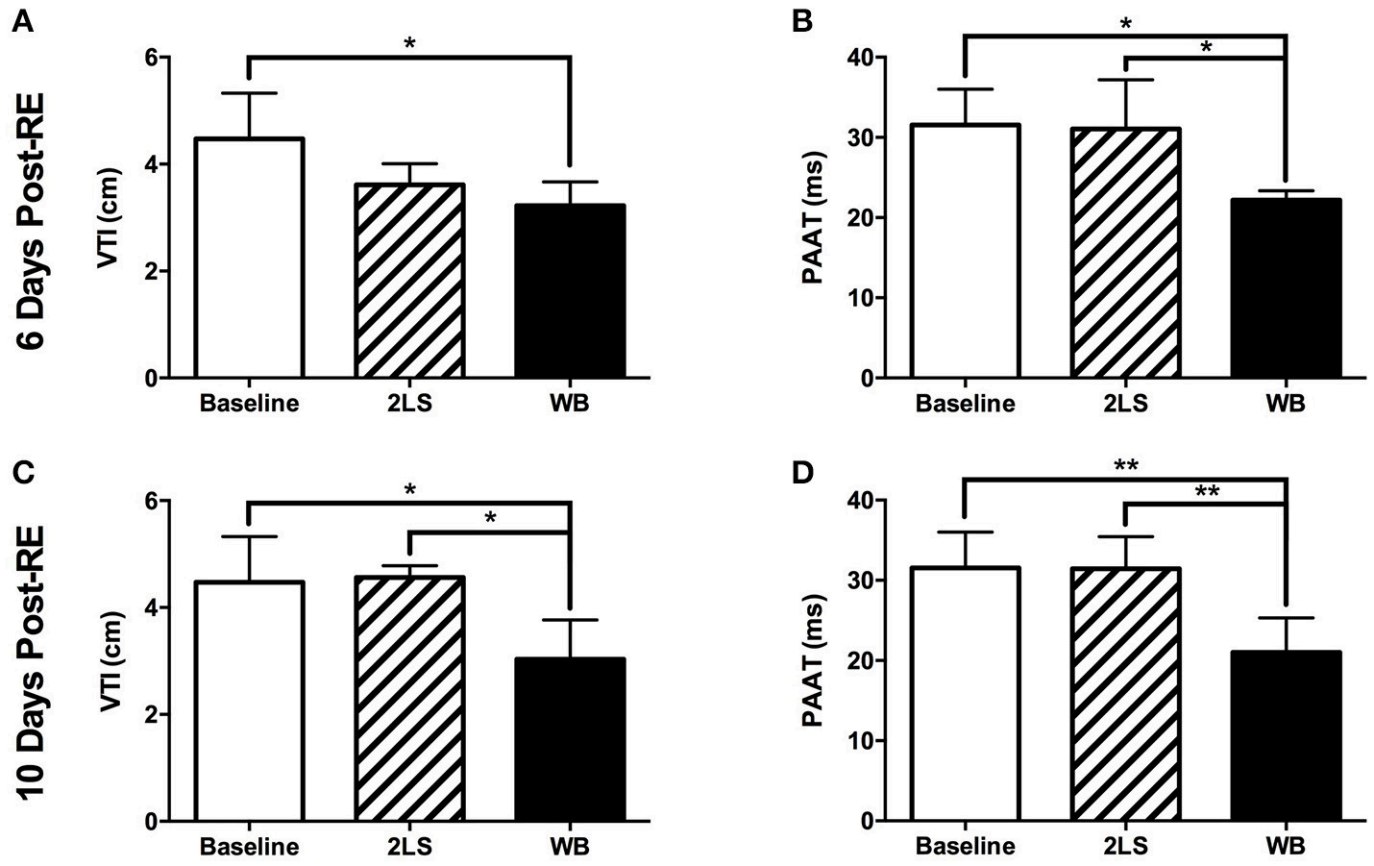
Acute effects of radiation exposure (RE) on pulmonary artery function. **(A,B)** Six days post RE, the velocity time integral (VTI) and pulmonary artery acceleration time (PAAT) were significantly decreased in the whole body (WB) irradiated group compared to baseline but only the PAAT was different from the 2-leg shielded (2LS) group. **(C,D)** Ten days post RE, VTI and PAAT were significantly decreased in the WB compared to baseline and 2LS groups. *N* = 4–7, ^*^p < 0.05, ^**^p < 0.01.

We investigated the effect of RE on the PA function by measuring PAAT and the VTI at baseline and in the WB and 2LS groups at days 6 and 10 post-RE. AT day 6 post-RE, we measured a significant decrease in both VTI (*P* = 0.0293) and PAAT (*P* = 0.0143) in the WB group compared to baseline, see Figures 3A,B. However, we see a significant decrease (*P* = 0.038) in PAAT in the WB group compared to the 2LS group, see Figure 3B. At day 10 post-RE, there was a still significant change in both VTI (*P* = 0.0114, *P* = 0.0184) and PAAT (*P* = 0.0028, *P* = 0.0081) in the WB group compared to baseline and 2LS (respectively), see Figures 3C, D.

### Blood results

**Figure 4:**
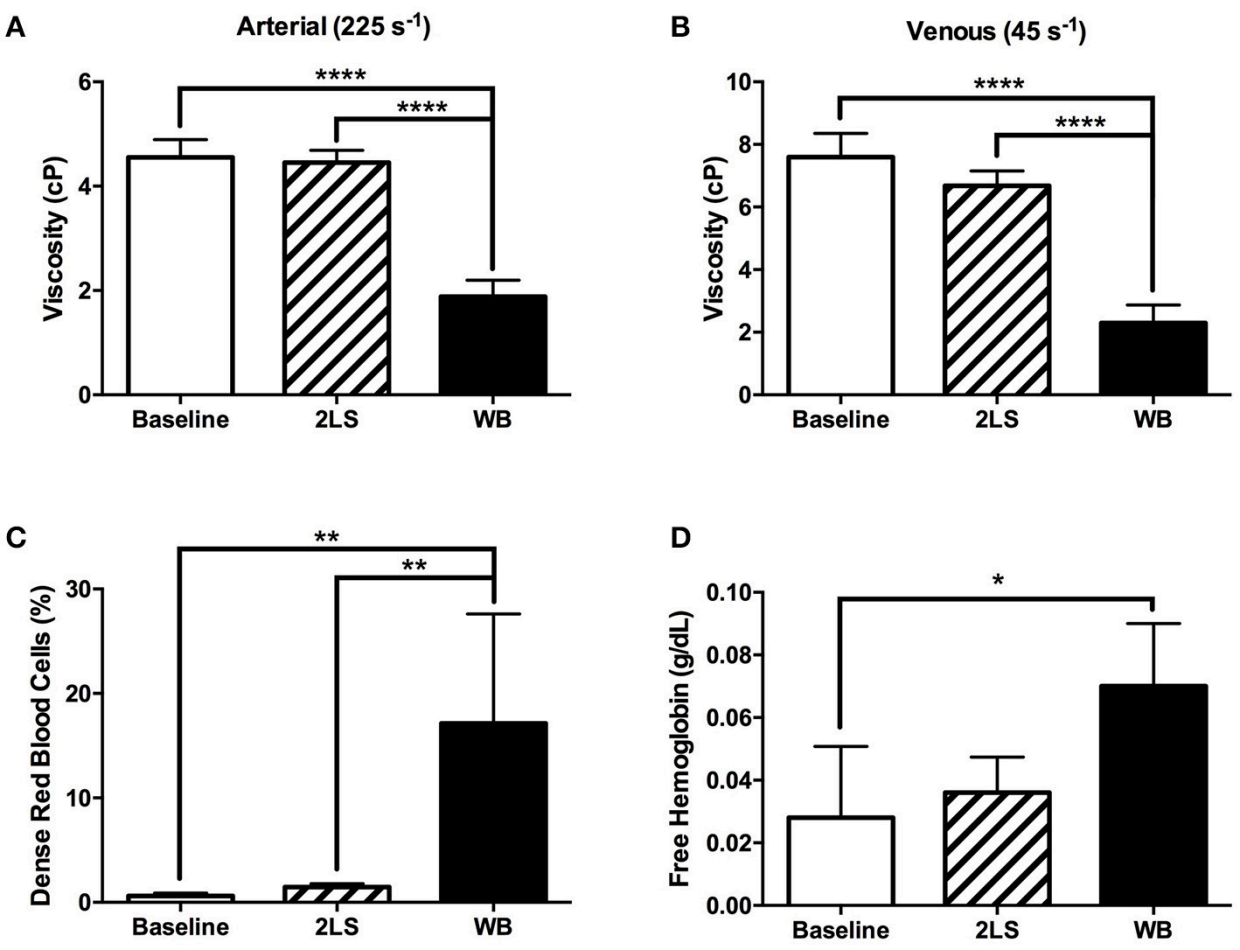
Radiation exposure (RE) of the whole body (WB), but not partial body (2LS), alters red blood cell rheology. **(A,B)** At 9 days post exposure, viscosity was measured at two shear rates 45s^−1^ and 25s^−1^, (venous and arterial circulation) and were significantly lower in the WB compared to baseline and 2LS animals, but not between the 2LS and baseline. **(C)** Percentage of dense red blood cells, a measure of “useless” erythrocytes, increased significantly in the WB compared to both baseline and 2LS, but 2LS was not different from baseline. **(D)** Free hemoglobin (total - cellular) adjusted for reduced erythropoiesis by dividing by total Hb was significantly higher in WB compared to baseline. *N* = 3–5, ^*^p < 0.05, ^**^p < 0.01, ^****^p < 0.0001.

We measured blood rheology following RE animals to investigate the relationship between RE on blood and cardiac function. Viscosity was measured at two shear rates 45s^−1^ and 225s^−1^, to mimic venous and arterial circulation, and were both found to be significantly lower (*P* < 0.0001 and *P* < 0.0001 respectively) in the WB compared to both baseline and to 2LS animals, (Figures 4A,B). Interestingly, the % DRBCs, was significantly higher in WB compared to baseline and 2LS animals (*P* = 0.0014 and *P* = 0.0027, respectively; Figure 4C). DRBC are more likely to hemolyze, and release free Hb; the rise in %DRBC with irradiation correlated with an increase in free Hb compared to baseline. Indeed, free hemoglobin was significantly higher (*P* = 0.0292) in WB compared to baseline.

**Figure 5:**
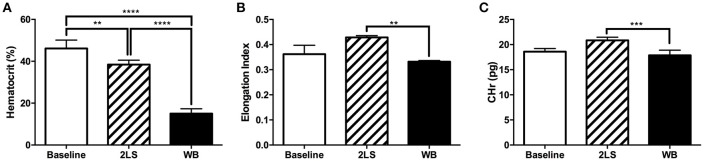
Radiation exposure (RE) affects rheological values. **(A)** At 9 days post exposure percent of hematocrit was significantly lower in whole body (WB) irradiated compared to baseline and two leg shielded (2LS) animals. The 2LS hematocrit was also significantly lower than baseline. **(B,C)** Red blood cell membrane deformability index and Reticulocyte hemoglobin content (CHr), a measure of the amount of hemoglobin in reticulocytes, both demonstrated a significant decline in WB compared to 2LS. *N* = 3–5, ^**^p < 0.01, ^***^p < 0.001, ^****^p < 0.0001.

We observed hematocrit changes at 9 days post-RE, the WB group showed a significantly decreased level compared to both the baseline and 2LS groups (*P* < 0.0001), and the 2LS group showed a significant decrease in hematocrit levels from that of baseline (*P* < 0.001; Figure 5A).The red blood cell membrane deformability index, or elongation index, and reticulocyte hemoglobin content (CHr), a measure of the amount of hemoglobin in reticulocytes, were both significantly lower (*P* = 0.0011 and *P* = 0.0005, respectively) in the WB compared to 2LS, see Figures 5B,C. Finally, the MCHC was significantly higher (*P* < 0.0001) in both WB and 2LS when compared to baseline and WB was significantly higher than 2LS, (Supplement Figure 1A). The MCV was significantly lower (*P* = 0.0003) in WB, but not 2LS, compared to baseline (Supplement Figure 1B). While the WB HDW, a measure of the heterogeneity of RBC hemoglobin concentration, was significantly higher (*P* = 0.0002) than both baseline and 2LS, (Supplement Figure 1C).

Further evidence of flow and oxygenation irregularities following RE was provided by the hematocrit to viscosity ratio (HVR), an indicator of oxygen carrying capacity (Supplement Table 3). HVR was calculated at two shear rates 45s^−1^ and 225s^−1^, to mimic venous and arterial circulation. In arterial flow HVR was significantly lower in both WB and 2LS compared to Sham (*P* < 0.0013, and *P* < 0.003, respectively). Hemoglobin levels were significantly lower in WB compared to both Sham and 2LS (*P* < 0.0001, for both). As expected, our measurements reproduced some of the hallmark changes in blood parameters such as decreases in white blood cell and platelet counts following exposure to WB and partial body radiation (Supplement Figure 2).

## Discussion

We have utilized a rat model of ARS in conjunction with cardiac US and MR imaging to demonstrate the utility of monitoring the cardio-pulmonary function as an early prognostic indictor to diagnose those that are more severely affected after RE. Our measurements could detect several significant differences between baseline and WB that were not in 2LS animals. We measured continual significantly lower VTI and PAAT at both 6 and 10 days and lower stroke volume in WB than sham/baseline levels, but not in the 2LS animals. We report significant changes in viscosity, %DRBC, free Hb, and RBC deformability in the WB, but not in the 2LS animals. Our findings support the use of CF and PAF as measures of the severity of radiation sickness and to a possible link between cardiac function and rheological changes post RE. However, a limitation of our study is that we did not identify a readily known cardiac syndrome that fits the observed radiation exposure effects but heart failure symptoms themselves are non-specific (15) and cardiac changes can be a result of several physiological, metabolic, and rheological causes.

PAAT has been shown to be a good indicator of pulmonary arterial hypertension (2, 16), and in our ARS model we demonstrated that PAAT function deteriorates as early as 6 days, with no recovery at 10 days, post-WB RE (Figure 3). The lung is a particularly radiosensitive organ, which can affect cardiac function. Gillette et al. (17) reported that localized 12Gy to the lung and heart resulted in reduced stroke volume and an increase in pulmonary artery pressure 3 months post exposure attributed to later-term fibrosis. However, our measurements are acute before the onset of fibrosis but some degree of pulmonary vascular resistance could develop early post-RE due to the direct radiation effect on the lung vasculature such as vasoconstriction which we and others have shown (18, 19). However, our data may also imply heart failure as indicated by a significant decrease in VTI values (20, 21) or the possibility of an increased load on the heart, which is unsustainable, leading to the decrease in stroke volume and cardiac output as measured.

In the current work, despite the heart and lungs receiving identical levels of radiation, the WB group had to be euthanized at approximately 10 days post-RE, while the 2LS group survives. The main difference between the groups is in the sparring of the hind legs' bone marrow, which plays a role in the recovery of damaged organs and may be responsible for restoring 2LS animal's cardiac function. Our previous work have demonstrated compensatory proliferation of shielded bone marrow following RE bone marrow depletion (8, 9), as well as, the recovery of the gastrointestinal tract corresponding with an increase in bone marrow metabolism in partially shielded animals (22). However, bone marrow sparing also significantly effects several rheology parameters (Figures 4A,B; Supplement Table 3) which could affect cardiac function. A significant decrease in viscosity in the WB but not the 2LS animals compared to baseline was measured (Figures 4A,B). This might, in part, explain the significant increase in ejection fraction observed in the WB but not in the 2LS post RE (Supplemental Table 1). Interestingly, we only observed a significant increase in WB total percent dense cells (Figure 4C) and a decline in erythrocyte elongation index (Figure 5B). Deformability and aggregability of RBCs, is related to changes in blood viscosity, oxygen delivery, endothelial function, and flow resistance (23–25). The severe drop in viscosity as well as aggregability are hallmarks of acute anemia which can lead to vasoconstriction and an increase in cardiac output. However, in our WB animals we observed a decrease in cardiac output (Figure 2) which may be due to heart exhaustion (26). Blood viscosity affects shear stress, which in turn can cause changes in endothelial function through its effect on nitric oxide (NO) levels (27). In combination, RE can induce endothelial dysfunction via its effect on NO levels (28–30) and vascular topology and diameter (19). This is supported by the findings that RE increased the amount of free circulating extracellular Hb (Figure 4D). Free Hb scavenges nitric oxide resulting in endothelial damage (31, 32) and is known to contribute to vascular and cardiac dysfunction (33–35). These rheology factors combined can lead to an increase in peripheral resistance, pulmonary resistance and cardiac dysfunction (36, 37), which could in part explain the differences in cardiac function observed between the 2LS and WB irradiated animals.

Our studies indicate that EC combined with rheological parameters might be an excellent option to help triage ARS victims and identify patients in need of acute therapy. EC is probably the initial diagnostic imaging modality available in a mass causality scenario due to its accuracy, ease of use, availability, portability, and cost (15). In an emergency situation an echocardiogram that differentiates between those that are in severe danger (WB vs. 2LS) would result in immediate decision-making and the initiation of therapy.

## Ethics statement

This study was carried out in accordance with the recommendations in the Guide for the Care and Use of Laboratory Animals of the National Institutes of Health. The protocol was approved by the Institutional Animal Care and Use Committee (IACUC) at Baylor College of Medicine.

## Author contributions

TI, JZ, and MWG planned the experiments and prepared the manuscript. TI, CCK, and JZ performed the experiments and processed the data. TI processed and analyzed the imaging data. VS, CCK, CeK, AP, and PS contributed to experiments. TI, and JZ performed statistical analysis and created manuscript figures. TI, JZ, VS, OS, and MWG all edited the manuscript.

### Conflict of interest statement

The authors declare that the research was conducted in the absence of any commercial or financial relationships that could be construed as a potential conflict of interest.

## Abbreviations

%DRBC: percent dense red blood cells
2LS: two-leg shielded
ACR: absolute reticulocyte count
ARS: Acute Radiation Syndrome
CF: Cardiac Function
CHr: reticulocyte hemoglobin content
EC: echocardiography
Hb: Hemoglobin concentration
HDW: hemoglobin distribution width
HVR: hematocrit to viscosity ratio
LVID: left ventricular inner dimension
LVOD: left ventricular outer dimension
MCHC: mean corpuscular hemoglobin concentration
MCV: mean corpuscular volume
MR: Magnetic Resonance
RBC: red blood cell count
RE: Radiation Exposure
PAAT: pulmonary artery acceleration time
PAF: pulmonary arterial function
VTI: velocity time integral
WB: whole body
WBC: white blood cell
