# Supplementary material for: Echocardiography Differentiates Lethally Irradiated Whole-Body From Partial-Body Exposed Rats
Source: Front Cardiovasc Med. 2018 Oct 16;5:138. doi: 10.3389/fcvm.2018.00138 (PMC6232677; doi:10.3389/fcvm.2018.00138)
Supplement: Supplementary file 1 [file Presentation_1.PPTX]

## Slide 1
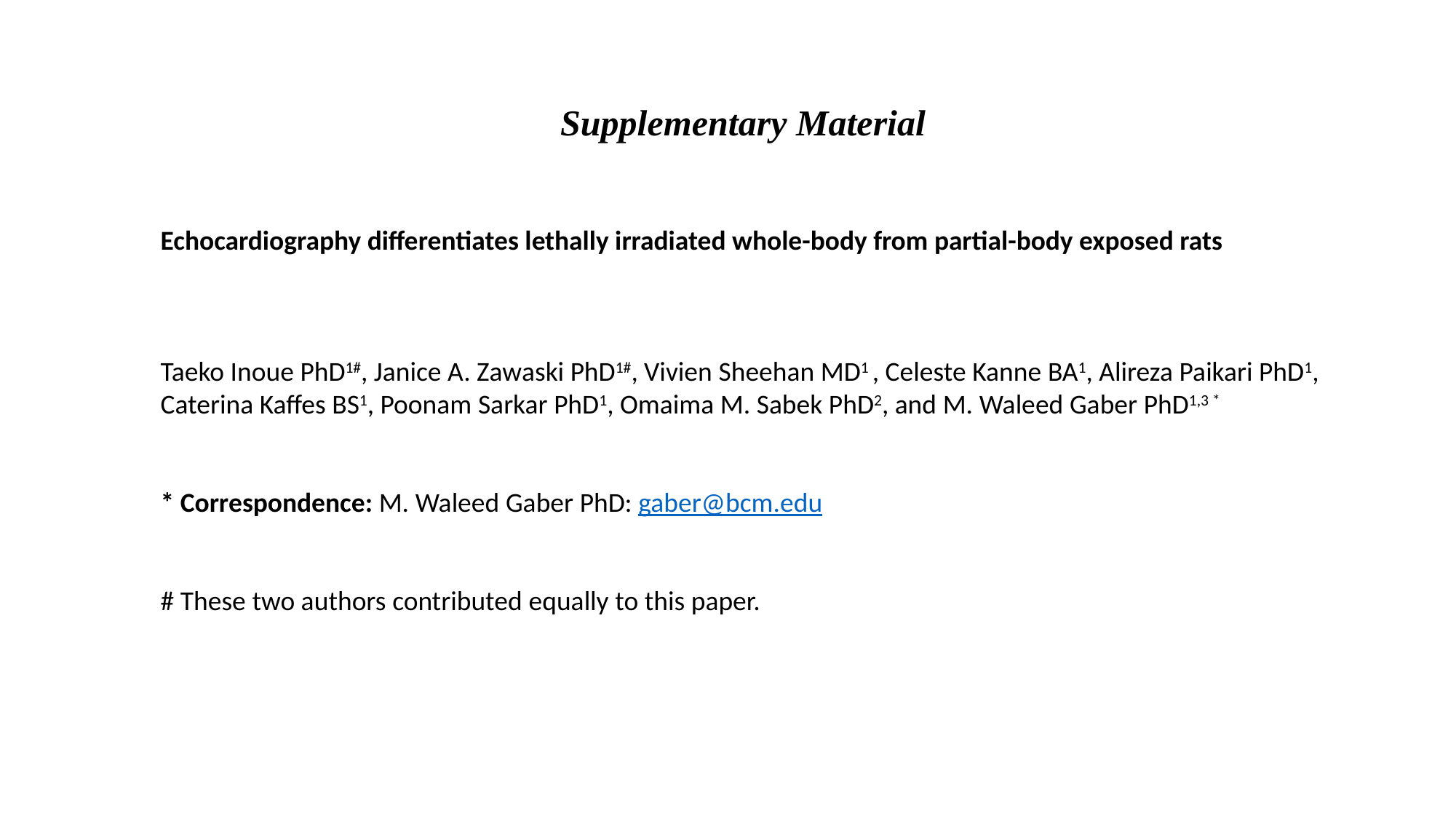

Supplementary Material
Echocardiography differentiates lethally irradiated whole-body from partial-body exposed rats
Taeko Inoue PhD1#, Janice A. Zawaski PhD1#, Vivien Sheehan MD1 , Celeste Kanne BA1, Alireza Paikari PhD1, Caterina Kaffes BS1, Poonam Sarkar PhD1, Omaima M. Sabek PhD2, and M. Waleed Gaber PhD1,3 *
* Correspondence: M. Waleed Gaber PhD: gaber@bcm.edu
# These two authors contributed equally to this paper.
#
1

## Slide 2
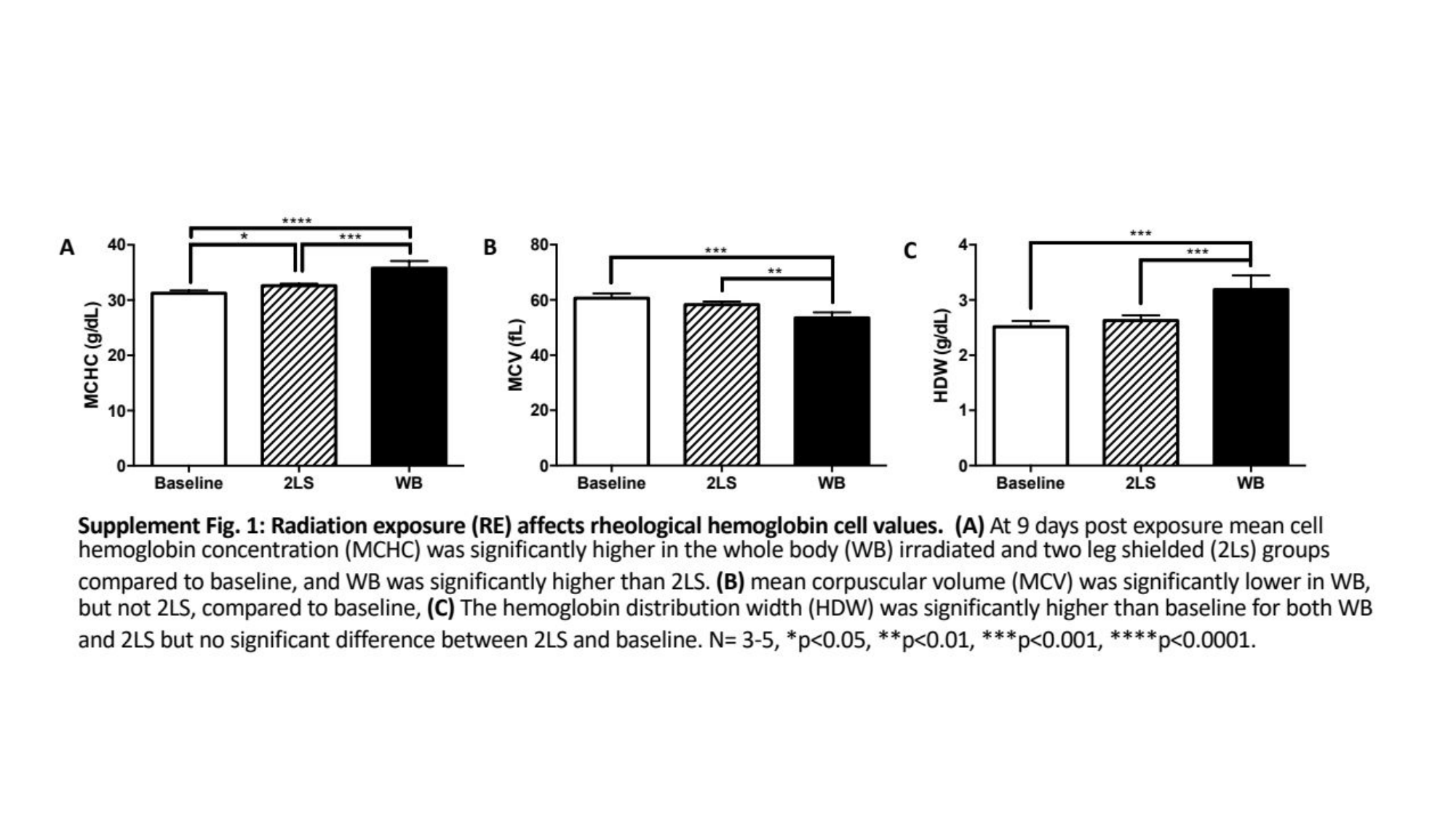

A
B
C
Supplement Fig. 1: Radiation exposure (RE) affects rheological hemoglobin cell values. (A) At 9 days post exposure mean cell hemoglobin concentration (MCHC) was significantly higher in the whole body (WB) irradiated and two leg shielded (2Ls) groups compared to baseline, and WB was significantly higher than 2LS. (B) mean corpuscular volume (MCV) was significantly lower in WB, but not 2LS, compared to baseline, (C) The hemoglobin distribution width (HDW) was significantly higher than baseline for both WB and 2LS but no significant difference between 2LS and baseline. N= 3-5, *p<0.05, **p<0.01, ***p<0.001, ****p<0.0001.
2

## Slide 3
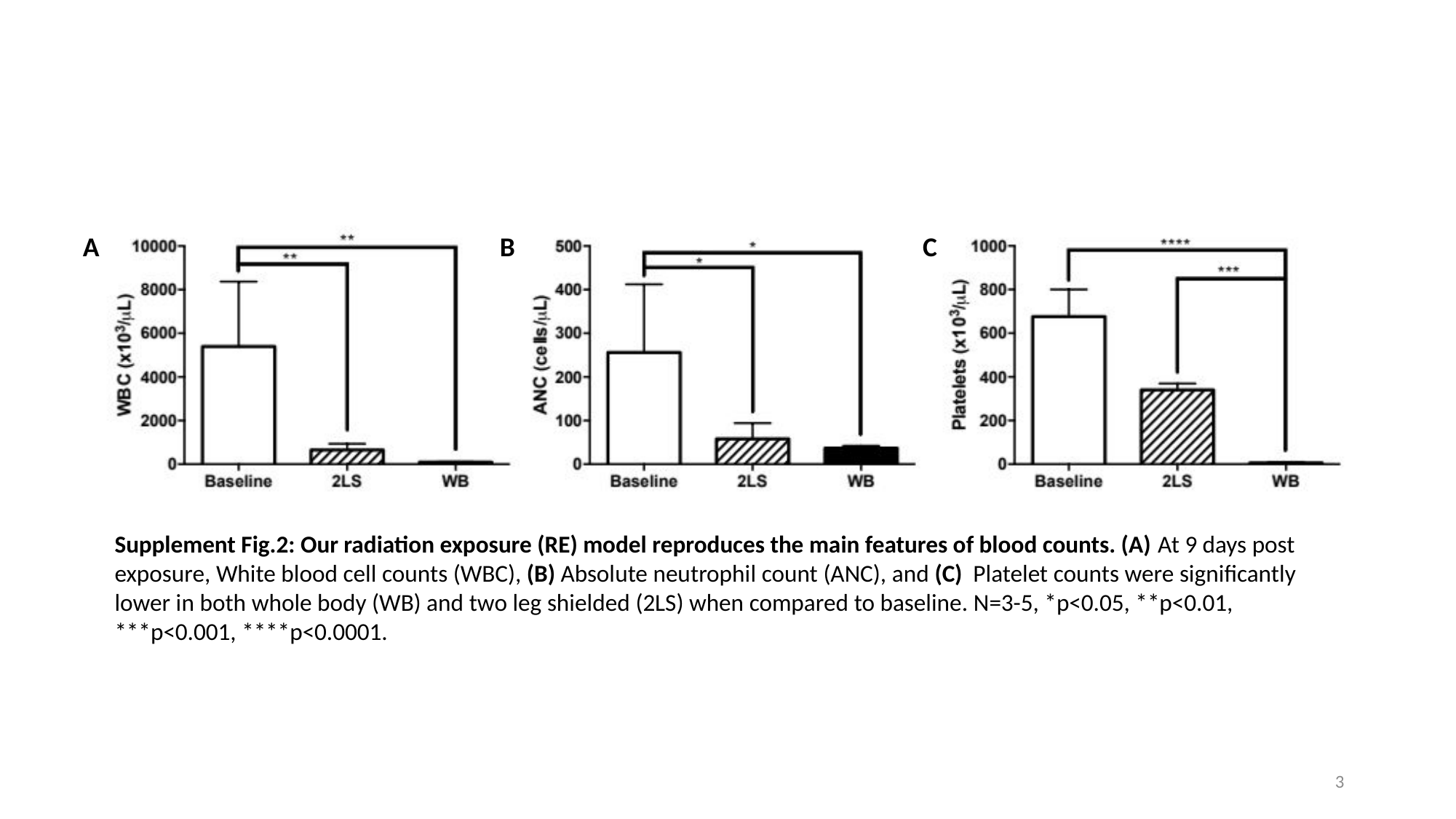

A
B
C
Supplement Fig.2: Our radiation exposure (RE) model reproduces the main features of blood counts. (A) At 9 days post exposure, White blood cell counts (WBC), (B) Absolute neutrophil count (ANC), and (C) Platelet counts were significantly lower in both whole body (WB) and two leg shielded (2LS) when compared to baseline. N=3-5, *p<0.05, **p<0.01, ***p<0.001, ****p<0.0001.
3

## Slide 4
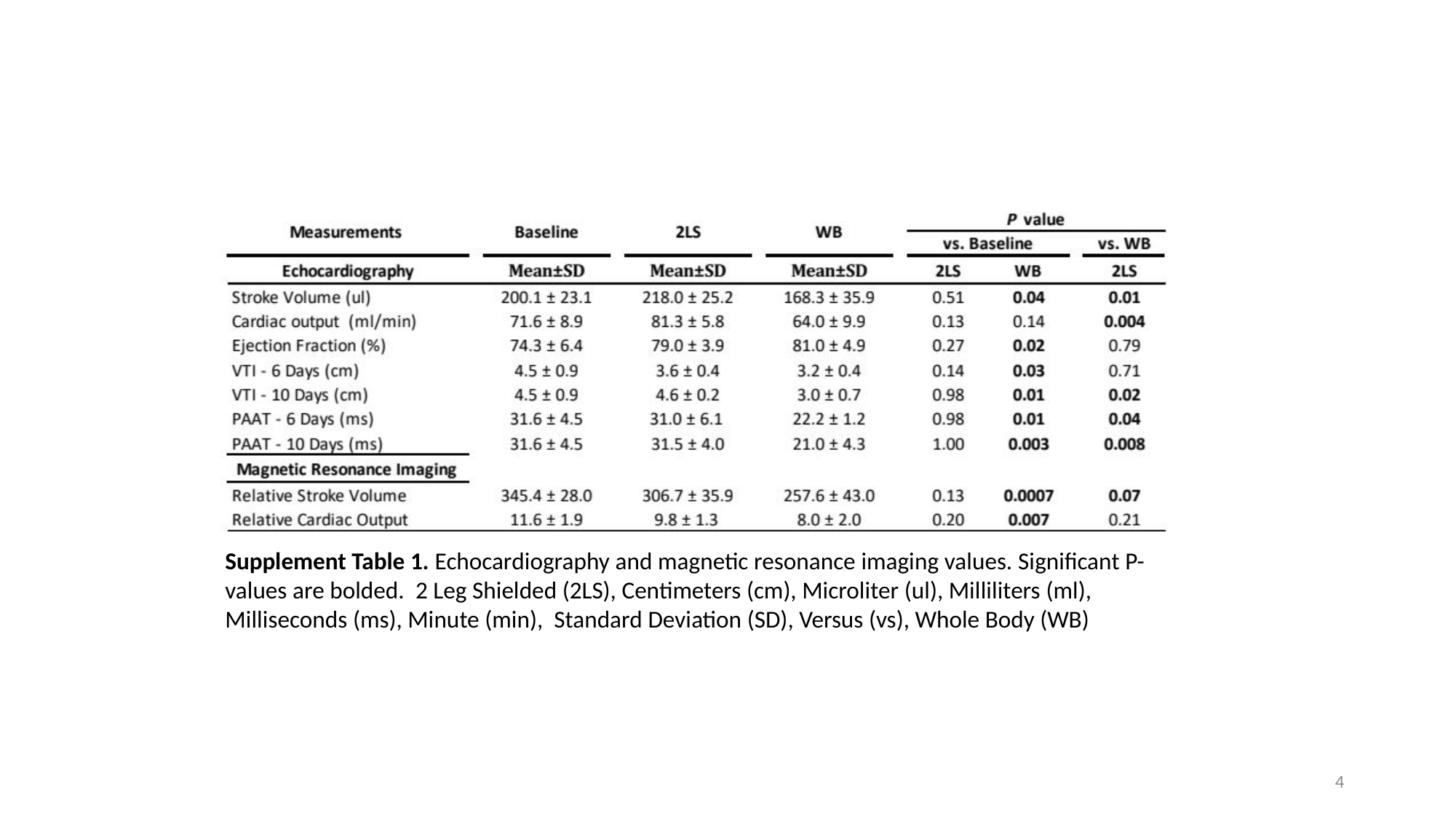

Supplement Table 1. Echocardiography and magnetic resonance imaging values. Significant P-values are bolded. 2 Leg Shielded (2LS), Centimeters (cm), Microliter (ul), Milliliters (ml), Milliseconds (ms), Minute (min), Standard Deviation (SD), Versus (vs), Whole Body (WB)
4

## Slide 5
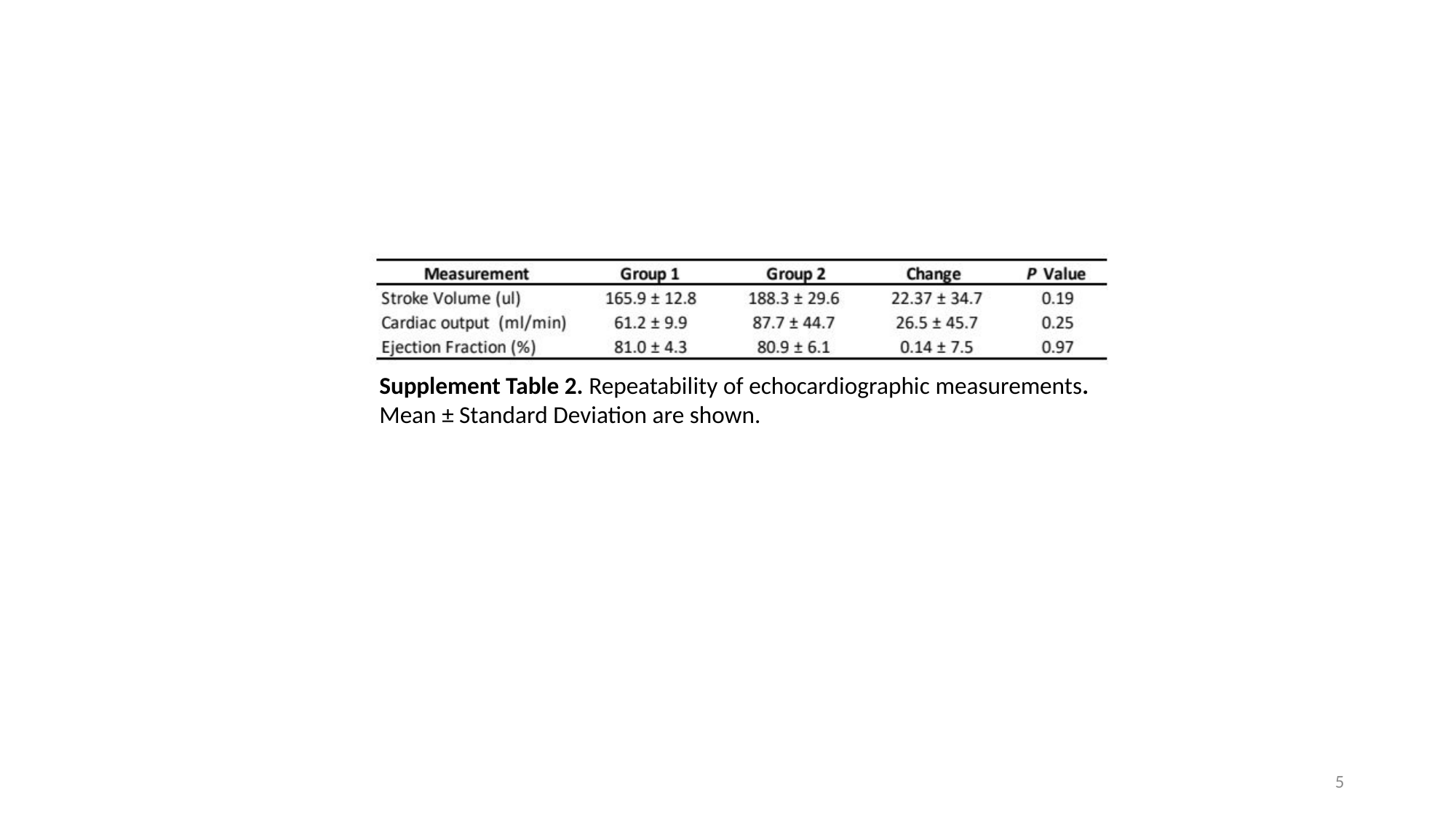

Supplement Table 2. Repeatability of echocardiographic measurements. Mean ± Standard Deviation are shown.
5

## Slide 6
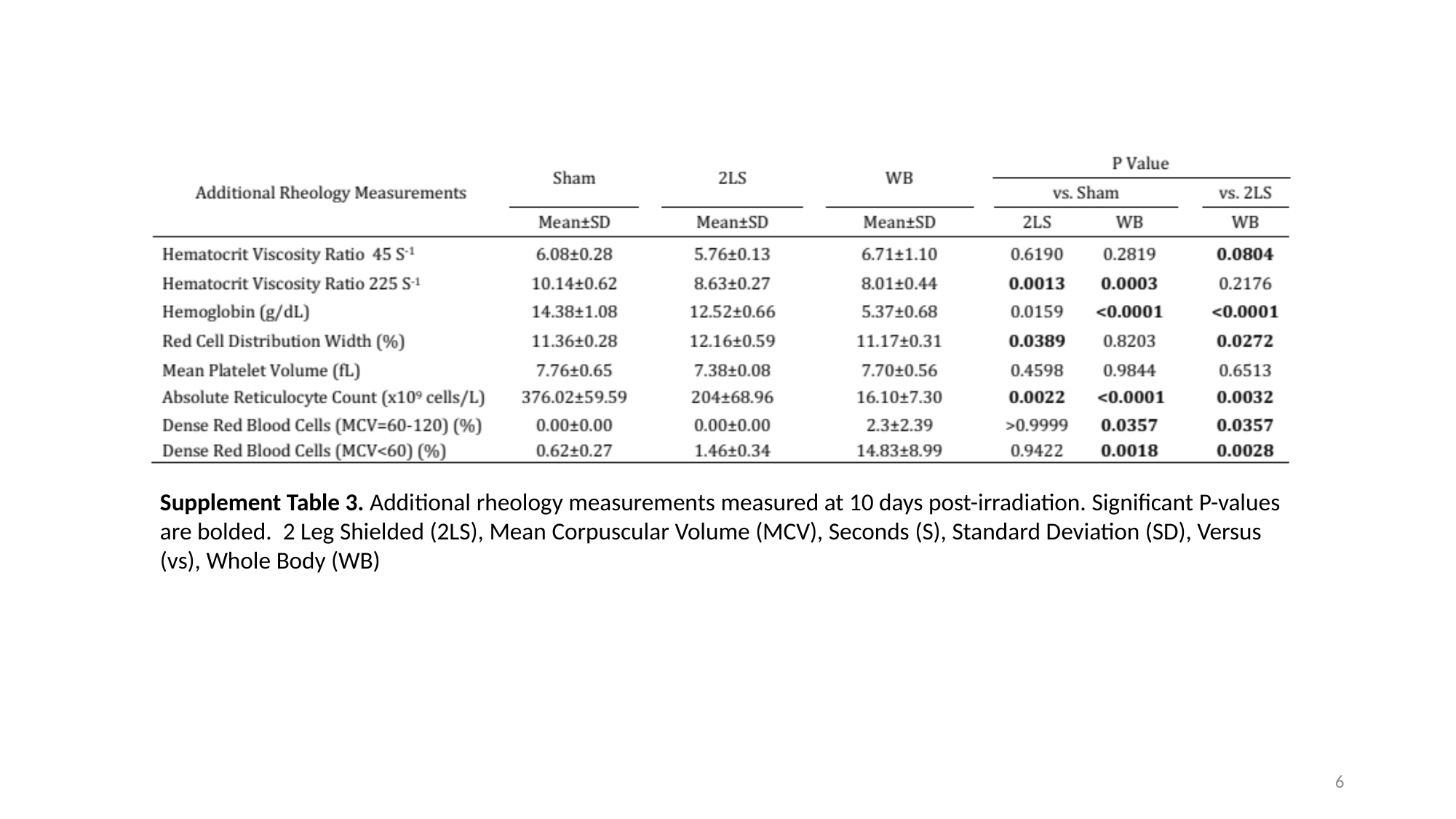

Supplement Table 3. Additional rheology measurements measured at 10 days post-irradiation. Significant P-values are bolded. 2 Leg Shielded (2LS), Mean Corpuscular Volume (MCV), Seconds (S), Standard Deviation (SD), Versus (vs), Whole Body (WB)
6
